# Forward-Looking Concept Functions and the Function/Accident Distinction

**DOI:** 10.1007/s10670-025-00957-1

**Published:** 2025-05-20

**Authors:** Aneta Zuber

**Affiliations:** Department of Philosophy, University of Zurich, Zürichbergstrasse 43, 8044 Zurich, ZH Switzerland

## Abstract

Appealing to the functions of concepts in conceptual engineering projects has been hailed as a promising avenue to explain defects of concepts, the possibility of amelioration, and the continuity of topic post engineering. Some of the most prominent theories of concept functions build on what I call the Usefulness Thesis, i.e. what makes something the function of a concept is what makes it useful. In this paper, I argue that this approach does not meet some of the most fundamental adequacy criteria for a theory of function, that is, (1) they do not draw a stable function/accident distinction and (2) they cannot account for the normativity of functions. I show that theories of concept function that build on the Usefulness Thesis are all essentially forward-looking theories of functions and show that these theories face a problem of overreach and a vacuous normativity proviso. I further argue that they do not lend themselves to conceptual critique, so they are not fit for conceptual engineering purposes.

## Introduction

It has been proposed in the conceptual engineering literature that ameliorating a specific representational device or concept consists of at least three steps:

Conceptual engineering (CE) =

(i) The assessment of representational devices,

(ii) reflections on and proposal for how to improve representational devices, and

(iii) efforts to implement the proposed improvements. (Cappelen & Plunkett, 2020)

In this paper, I want to focus on step (i), namely the assessment stage of conceptual engineering. One proposal for how to approach an assessment of our concepts^1^ has recently gained some attention. The proposal is: in order to understand and critically evaluate a particular concept, we first ought to understand its function. Several authors have already attempted to supply a theory on how to analyze concept functions (Prinzing, 2018; Thomasson, 2020, 2022; Haslanger, 2020; Queloz, 2021; Simion & Kelp, 2019; Nado, 2019; Jorem, 2022; Köhler & Veluwenkamp, 2024). These texts build on three potential desiderata for a coherent theory of concept functions^2^:

1. Assessment of concept efficiency
  Concept functions furnish standards to assess how well a concept fulfills a function e.g. If the function of RAPE^3^ is to refer to all and only objectionable occurrences of sexual violence, then we can inquire how well our concept of RAPE does that.
2. Topic continuity
  Concept functions provide a non-arbitrary connection between the pre-amelioration and post-amelioration concepts. e.g. RAPE: If we preserve the concept functions, then the pre-amelioration and post-amelioration concepts have something essential and principled in common.
3. Amelioration direction
  Concept functions can direct us in ameliorating our concepts e.g. RAPE: If the function of rape is to refer to all and only objectionable occurrences of sexual violence and it does not yet do that, then we should ameliorate it to capture all of them.

Most contributions to this debate only investigate concept functions insofar as they can deliver at least one of these three desiderata.

What I call the Usefulness Thesis is thought by some (see Sect. 3) to be a way to build a theory of concept functions that fulfills these desiderata. What is the Usefulness Thesis (UT)?

(UT) Usefulness is (partly) determinative of a concept’s function.^4^

This means that a concept’s function is, at least in part, the usefulness of the concept for its users (or its usefulness in a community at large). X being useful in this context is to be understood as the state of enabling x to fulfill a *practical purpose*; meet a need, or achieve a desired outcome y effectively. This connects to conceptual engineering’s goals to engineer effective concepts that make a practical difference, i.e. ‘engineering’ as ‘problem-solving’ (Isaac et al. (2022)). When it comes to ascribing a function to a concept, it is necessary to have a clear grasp of the concept’s usefulness. The reason for this trend is that it is broadly accepted that concepts were not designed to do something specific in the same way that artifacts are, but simultaneously concepts are often branded ‘tools’or ‘devices’in the conceptual engineering debate. This suggests proximity to artifacts. Concepts are after all used and, broadly speaking, created by specific communities. There are good reasons to think of concepts in terms of their practical contribution.

In this paper, I intend to take a step back. Rather than starting with the three traditional desiderata and constructing a theory of concept functions based on them,^5^ I will embark on a propaedeutic project. Specifically, I will question whether the accounts that build on (UT) and the aforementioned desiderata truly capture the contribution of the function of a concept that is relevant to conceptual engineering. In other words, I will examine whether the prevailing theories in conceptual engineering are suited to fulfilling their intended roles. While I support the functional turn in conceptual engineering and, like many other conceptual engineers, believe that recognizing the functional nature of concepts is crucial to the field, we still need a clearer understanding of which account of function serves our aims.

I will argue that the Usefulness Thesis leads to accounts of concept functions that are not explanatorily stable and impede conceptual critique necessary for conceptual engineering purposes. I will do this in three steps. First, I will introduce a canonical taxonomy of theories of function. Second, I will spell out why etiological theories of concept functions have subsided in the debate. Then, I introduce the origins of the Usefulness Thesis. For one, it has its roots in the rejection of etiological functions for the purposes of conceptual engineering. For another, there is a trend toward functions that can give normative standards to conceptual engineering projects and serve the concept users which has led to two prominent concept function accounts. The theories that build on (UT) are shown to be a sub-type of forward-looking theory of function. I will argue that concept functions as understood in terms of (UT) inherit certain widely recognized problems from forward-looking theories in terms of explanatory power. Further, I will argue that they do not lend themselves to conceptual critique because they are inherently justificatory accounts that gloss over conceptual shortcomings.

## Theories of Functions

The debate about functions and how to analyze something functionally has a long tradition in the philosophy of biology. Questions about why organs, such as the heart and kidneys, could have functioned without someone having intentionally designed them, have led to an explosion in different theories of functions. Theories of functions that attempt to give an answer to these questions are standardly divided into three broad camps: backward-looking accounts, forward-looking accounts, and presentist accounts of functions (see for example Garson (2007)). I will very briefly introduce each account by giving a general definition of a sort of theory and then how it has been developed to explain the function of traits of certain organisms.

First, backward-looking accounts, also commonly called etiological accounts of functions, posit that the function of a trait is what it has been selected to do (Wright, 1973; Neander, 1991a, b; Millikan, 1984, 1989; Griffiths, 1993; Godfrey-Smith, 1994). According to this view, traits that have been favored by natural selection are those that have historically contributed to an organism’s reproductive success, ensuring their presence in future generations. In other words, the function of a trait is the causal contribution that makes a difference to the organism’s survival. For example, the heart has the function of pumping blood because its causal contribution, to transporting nutrients and removing waste, has proved beneficial to animal survival.

Next, presentist theories of functional analysis focus on the contribution of a trait or subsystem that it has now. These are called causal role or system function theories (Cummins, 1975; Preston, 1998). A trait can have different functions relative to the system specified (for discussion see McLaughlin (2000)). Cummins argues that “a capacity of a containing system is appropriately explained by analyzing it into a number of other capacities whose programmed exercise yields a manifestation of the analyzed capacity, the analyzing capacities emerge as function” ((Cummins, 1975), p. 765). In other words, if a pertinent system has certain capacities (for example, a specific assembly line has the capacity to produce phones), then this capacity is constituted by its subsystems and their capacities (here, the design department, the production of material parts of the phone, the order in which the phone is to be assembled, etc.); if the subsystem and its capacities causally contribute to the capacity of the larger system (again, to produce phones), they can be said to have a certain function relative to the system. This is useful for biological traits, for example, if a new gene mutation has certain causal effects on the body, this is where the causal accounts shine. Etiological accounts are not well-equipped to explain the very first instances of a trait or spontaneous mutations since these traits do not yet have a history of selection. Therefore, a focus on the present contributions of the trait can be very useful to model new causal contributions and explain their function.

Forward-looking or consequentialist theories are concerned with the *positive effects* that the trait has on the system’s future. Unlike backward-looking theories, forward-looking ones bracket what purpose a trait has fulfilled before and only attend to the trait’s *positive* contribution. There are several ways of specifying which ‘positive’ contribution is relevant for the function and what it is a trait is supposed to contribute to. There are several influential sub-types of forward-looking accounts. Firstly, there are good-contribution theories. An effect of a trait can be said to be the function of the trait if and only if the organism benefits from it. Secondly, others advocate for fitness-contribution theories of function. Behind these accounts is the simple idea that some effects contribute to an organism’s fitness (Lehman, 1965; Ruse, 1971, 1973; Bigelow & Pargetter, 1987; Horan, 1989; Walsh, 1996; Wouters, 2003). Next, there are goal-contribution theories that allow function ascription to the parts of an organism if they contribute to the goal of the system (Boorse, 1976). The idea is that any system has a goal if it displays a capacity to behave flexibly and persistently to achieve a certain goal ((Nagel, 1977), p. 272). Self-organizational accounts, such as Maturana and Varela (1991), Mossio et al. (2009), and McLaughlin (2000) belong to that ilk. I will draw on this taxonomy in the rest of the paper.

## Etiological Functions of Concepts

One reason why the Usefulness Theses (UT) I introduced above is becoming more popular is that etiological theories have arguably failed to provide a serviceable account for conceptual engineering projects Riggs (2021); Jorem (2022). Etiological theories of functions posit that the function of a concept is what it has been selected to do by either natural or psychological mechanisms or processes (excluding intentional design) (Simion & Kelp, 2019; Millikan, 1984; Prinzing, 2018). I want to summarize the current trend of moving away from the etiological theory as the most promising one to analyze concept functions.^6^

First, let’s look at the currently most influential formulations of an etiological account of concept functions developed by Simion and Kelp (2019). Simion and Kelp define the etiological functions (e-functions) of concepts in the following way:

A token of type T has the e-function of type B of producing effect E in system S iff

- tokens of T produced E in the past;
- producing E resulted in benefit of type B in S/S’s ancestors; and
- producing E’s having B-benefitted S’s ancestors contributes to the explanation of why T exists in S. (Simion and Kelp (2019), p. 263))

The first major issue facing etiological theories is that they have an epistemic access problem. The processes and mechanisms that have ensured a concept’s survival are extremely multifarious (see also (Riggs, 2021). Etiological theories of biological traits have the advantage that they need only take into account the natural environment in which a trait has developed and its recent history. However, it is by no means easy to determine all parameters for the etiological analysis of concepts because they are not only beholden to our natural environment, but also our psychology, history, technological as well as social development, and whatnot. The functions of biological traits and the functions of a concept are at least not prima facie analogous. A lot more theoretical and empirical work would have to be done to disentangle the factors that go into concept selection.^7^ If it is insurmountably difficult to trace how an effect of a concept has been selected, it is also insurmountably difficult to know whether something is a selected effect or not. The factors that contribute to a concept’s survival are for the most part epistemically inscrutable.

The second issue is that etiological theories of concept functions have an indeterminacy problem. Justin Garson introduces the problem as follows:

The heart beats. In beating, it moves blood around the body. This brings nutrients to cells and eliminates waste-essential activities for keeping us alive. Which of these activities, precisely, is its function? Are all of these activities its functions, or just one? And if only one, which? Does it matter? ((Garson, 2019), p. 109)

The problem with etiological theories is that they are not by themselves able to give us an answer to which of these functions is the proper one. Due to the fact that the heart beats, by doing this, it moves blood around the body, and thus brings nutrients to its cells and eliminates waste-all of these effects were selected for (Garson (2019), p. 110). Is the proper function of the heart a proximal function (like beating) or a distal function (such as moving nutrients and eliminating waste)? Why prefer one or the other and why not all of them? This is a reputed problem for etiological theories of function (for different versions of this objection in the context of teleosemantics see (Neander (2017), ch. 7)).

The same problem applies to etiological concept functions. Before I get into the problem, it should be noted that one major desideratum for a concept function account is that it should deliver invariantist rather than variantist concept functions (Cappelen (2018), p. 182).^8^ This means that the function of a concept should not vary but remain stable across varying (sentential and other) contexts. If concept functions should deliver, for instance, topic continuity, then concept functions should generally be stable through the process of semantic change. It has however been noted by philosophers opposed to the idea that concept functions can deliver topic continuity that no concept functions account is able to determine invariable individuation conditions of concept function descriptions (Belleri, 2021; Jorem, 2022).^9^

Take the concept of MARRIAGE. The concept, and thereby the institution, of MARRIAGE has gone through big changes. Before the 19th century,‘marriage’was used to mark certain heteronormative relationships worthy of protection, to manage economic resource allocation by facilitating a gendered division of labor, etc. Gradually, after the 19th century, our concept has changed to a less heteronormative and romantic notion. Can we identify an invariable function throughout these changes? Is the correct level of analysis more proximal (e.g. the function is to impose heteronormative rules, clarify inheritance) or distal (e.g. the function is to structure domestic life or to help us satisfy basic human needs)? Even if both the proximal and distal levels of analysis are correct, which level of function description is the correct one to assess the concept’s efficiency, conserve topic continuity, and guide the direction of amelioration? There is no obvious answer that proponents of etiological concept functions like Simion and Kelp can give.

The last issue with etiological theories of concept functions is that they lack ‘authority’for conceptual engineering projects (Queloz, 2022). Even if it is true that a specific concept has been selected for, there is no guarantee that the reasons and causes that contributed to that selection are any more relevant to our purposes and reasons now. Again, given the plausible idea that concept functions should give us reasonably determinate function ascriptions across contexts (the invariantist commitment) and that a concept functions account should deliver concept assessment, topic continuity, and the direction of amelioration, etiological theories of function cannot play that role. Queloz insists that “[j]ust because a concept’s past effects contributed to its being around now does not necessarily mean that we want to see these effects realized in the future.” The problem is that although concepts may have something akin to proper functions (the point of the authority objection is not to deny this), they are not the relevant functions that are conducive to ameliorative projects of our conceptual repertoire. If concept functions are to provide any of the three desired things mentioned in the introduction, then conceptual engineers should not build on etiological functions. Consider for example ideological concepts that help to reproduce oppressive systems (e.g. HYSTERIA, SEXUAL DEVIANCE, BLACK/WHITE, WOMAN/MEN): “To someone who thinks that the history of our concepts is pervaded by dynamics reproducing the patriarchy, engineering for e-functions points precisely in the wrong direction” ((Queloz, 2022), p. 16).

I am not fully convinced that etiological functions have been given the final death blow and have been definitively proven to be useless to conceptual engineers.^10^ But there has been a definitive shift in the conceptual engineering literature away from backward-looking accounts to forward-looking accounts with the promise that they furnish conceptual engineers with a better, more useful, understanding of concept functions. In the rest of the paper, I want to argue that we should be critical of one version of this shift. This argument is not supposed to mean that we should abandon the idea that concept functions are substantive tools in the conceptual engineer’s toolkit or that we *should* support etiological functions instead. Traditional theories of functions-the etiological and system functions accounts-have been under fire in favor of newer accounts, among them the accounts that are built on the Usefulness Thesis. This paper does not aim to give a positive argument for either of the two traditional theories of functions but instead aims to assess this new trend.

## Origins of the Usefulness Thesis

The Usefulness Thesis appears in two recent influential accounts of concept functions in conceptual engineering. Let’s look at them in turn. The first instance is hinted at in the writings on concept functions by Thomasson (2020).^11^

Thomasson recognizes that [identifying the functions of concepts] is hard, but we can get a‘set of clues’by asking: What makes the linguistic items in question useful for us? In particular, we should ask “what we can do better with such a vocabulary than if we lacked it” (Thomasson cited in (Cappelen (2018), p. 186)).

Thomasson then describes the process of analyzing concept functions in the following way:

[O]ne proceeds first by investigating what it does and can do, and thereby gains clues to determine what (system) function(s) it serves and how it serves them. So similarly, in engaging in conceptual engineering one may aim to engage in reverse engineering the concepts in question-aiming to determine what they do or can do-why it is useful to have such (ranges of) concepts at our disposal, what we can do better with such a range of concepts than if we lacked it. ((Thomasson, 2020), p. 448, emphasis added)

Although Thomasson has not yet developed this very specific thought,^12^ it seems clear that she thinks that the way a conceptual engineer should approach a conceptual engineering project is through the lens of the concept’s usefulness. The way to arrive at a functional ascription for a concept is to consider what we would miss if we lacked that concept. Let’s call Thomasson’s view the Absence-Consideration Account. The features that considering the absence of a concept ought to identify are the ones that are useful and thus enable us to do certain things more effectively or efficiently. Thus, it builds on the tenet of the (UT).

The most developed account, that explicitly cashes in on the (UT) as a substantive thesis, is Queloz (2022) Concern-Satisfaction Account of concept functions.^13^ The motivation behind this account is Queloz’s rejection of the etiological account of concept functions based on the Authority Problem expounded above and its relevance for conceptual engineering. He proposes that “[i]nstead of scrutinizing history for mind-independent causal patterns of carving up the world into impersonal systems, [we should examine] which among the effects of using a concept^14^ tend to tie in with human concern in a way that is conducive to their satisfaction” ((Queloz, 2022), p. 19). Thus, we should be analyzing the effects of our practice of concept use. The function of a concept that can be identified by this method is called a concern-relative function (‘c-function’). More formally, he defines c-functions as follows:

Concept X has the c-function of type C of producing effect E iff

- users of X have among their present concerns-their needs, interests, desires, projects, aims, and aspirations-a concern of type C;
- under propitious circumstances, applications of X produce E;
- E stands in an instrumental relation to the concern of type C, which is to say that under propitious circumstances, producing E contributes to the satisfaction of a concern of type C.

This needs clarification. First, this account is not about a single concept application but about “the concept’s effects across a wide range of contexts” ((Queloz, 2022), p. 19). If a particular concept’s application generates some beneficial effect, this does not mean that this application is the function of that concept. Instead, we ought to examine “whether [the concept’s application] *reliably correlates* with the satisfaction of certain concerns” and in which ways the “concept manifests the power to make a useful difference to human affairs” ((Queloz, 2022), pp. 19–20, emphasis added). Second, Queloz stresses that the effect E produced by the application of C does not need to be uniform ((Queloz, 2022), p. 19). Not all applications of the concept will have the same effects. He clarifies that “[t]o claim that concept X has a c-function of type C is to claim only that when circumstances concur, applications of X satisfy a concern of type C by producing E”; thus, for the sake of analysis, we initially bar “unpropitious circumstances, backfiring uses, and deviant causal chains” ((Queloz, 2022), p. 19). Third, the concept’s function is not about producing effects that are beneficial for everyone and all things considered ((Queloz, 2022), p. 30).

This account has several advantages. The first advantage is that the Concern-Satisfaction Account promises to specify functions of concepts that have not yet been fulfilled. Because a concept’s function is any effect that it would produce by application to make a difference to human concerns,.^15^ it is not restricted to actual effects of concept use but can take into account potential uses. For instance, if concept X can satisfy a concern that has emerged due to a new technical advancement, X may take on a new function.^16^ Next, it better explains how concepts can have any authority over us. Queloz develops a “service conception of conceptual authority” which dictates that “a concept’s claim to authority over our lives is vindicated if and to the extent that it demonstrably serves, or equivalently, ties in with a concern we would endorse upon reflection” ((Queloz, 2022), p. 21). Thus, a concern gives us a reason for concept use. The relation between our concerns and the reasons that figure in our reasoning is indirect (Queloz, 2022, p. 21). Queloz gives an illuminating example. Given our concern about avoiding danger, we have reasons to use the concept FIRE (e.g., to identify fire, tell others about fire, etc.): the presence of fire usually means danger, so we have reasons to apply the concept FIRE, and this application, we have reasons to avoid whatever FIRE applies to (Queloz, 2022, p. 22).

Given the above taxonomy of theories of biological function, it seems clear that Queloz’s Concern-Satisfaction Account is a forward-looking theory of function applied to concepts. As shown in the above taxonomy, there are many forward-looking effects and capacities of an explanandum that could constitute a function of the explanandum; this means that there are different ways to understand which ‘positive effects’ are the relevant ones for our theoretical purposes. Though the Concern-Satisfaction account is not identical to fitness-contribution, it is very close to a or goal-contribution theories or a good-contribution theory. Queloz’s theory promises to identify the effects of a concept that “reliably [correlate] with the satisfaction of certain concerns” and “manifest the power to make a useful difference to human affairs” (Queloz, 2022, pp. 19-20). In other words, this theory focuses on the effects that a concept has now, and how they tie in with and contribute to satisfying the concerns that concept users have and will have in the future as well as their practical contribution. Even though Queloz himself does not position himself with these theorists explicitly, he does seem to be aware that his account is allied with them. He emphasizes the fact that his account is “prospective and evaluative” and that it “looks forward to how concepts promise to earn their place in our repertoire as we meet the future” (Queloz, 2022, p. 23, my emphasis).^17^

The Concern-Satisfaction Account is promising and has a few advantages over other accounts, such as its emphasis that we use concepts to structure the world, that we have reasons for certain concepts rather than others, and the plausible idea that we have some control them.^18^ However, despite its advantages, I will argue in the rest of the paper that accounts that draw on the (UT) are too explanatory unstable for they identify too many effects with a concept’s function, and that they are uniquely disadvantaged in giving critical analyses of concepts.

It is important to note that forward-looking theories are diverse in several respects.^19^ Specifically, the theories that define the function of x as its capacity to contribute to fitness, and those that describe it as the capacity to contribute to need-satisfaction, do not identify the same capacities or effects. Similarly, the Concern/Need-Satisfaction and Absence Consideration accounts represent only a subset of forward-looking theories. What unites these accounts under the broader category of forward-looking theories is their shared focus on identifying a concept’s positive contribution to future outcomes. There has been a recent effort to develop a forward-looking theory that moves away from the Usefulness Thesis-namely, the Normative Functions account proposed by Köhler and Veluwenkamp (2024). I will briefly address this account in the final section. For now, I will focus on the subset of forward-looking theories grounded in the Usefulness Thesis.

## Problems With Forward-Looking Usefulness Theories

Forward-looking theories, however, face several issues.^20^ In this section, I want to investigate whether the Concern-Satisfaction account inherits these problems from the forward-looking theories of function.^21^ Note that the Concern-Satisfaction account need not necessarily have to carry over these problems, because we are switching the explanandum from biological traits to concepts which may change the analysis landscape somewhat. Nonetheless, I will argue that they give very unstable explanations of concept functions and thus fail on explanatory power.

The problems that forward-looking theories are usually confronted with are that they potentially violate two basic desiderata for theories of functions, i.e. the function/accident distinction and the normativity of function. Failing to meet the two desiderata curtails their explanatory power. Since these desiderata apply to functions generally, and not only to concept functions, they are more fundamental than the desiderata listed in the introduction. There is further reason to doubt whether the three desiderata mentioned in the introduction are sufficient to guide us to the best concept functions account. Firstly, it has been argued that nothing can satisfy all three desiderata together (Riggs, 2021). I have some reservations about this argument because, for example, Köhler and Veluwenkamp (2024) show that it is possible for a theory of function to at least *address* the three desiderata. I agree with Riggs that not enough work has been done to show how these desiderata hang together and therefore we should take seriously that there can be significant tension between them.^22^ Secondly, it is conceivable that there could be an account of concept functions that in some way satisfies the three desiderata but is not explanatorily stable, i.e. it does not satisfy the function/accident distinction or the normativity of function. This account would be undesirable for conceptual engineering purposes: if we commit to problem-solving a conceptual deficit, we should not base our suggestion on a side effect and sideline the ‘core’ functions, so to speak, of a concept. For example, imagine a concept that picks out a certain natural kind, but it has also an effect to cause suffering to a community because of certain additional features. One could imagine an account of concept functions that would pick out these effects, in order to identify strong reasons for the community to deal with the harm caused. (see Simion (2017) for a discussion of this problem) However, at the very least, the right theory should make us aware that the main purpose of the concept is to pick out a natural kind, and if we tinker with the concept based on a functions account that prioritizes harm, then we could lose a core purpose of the concept. Further, the two desiderata I propose here are *necessary* for the three desiderata specific to conceptual engineering. For example, to maintain topic continuity, we need a comprehensive explanation of the normativity of function that distinguishes between what a concept is supposed to do and what is a mere accident. This distinction can ensure topic continuity only if it remains consistent across various contexts (Cappelen, 2018). For these reasons, I take desiderata concerning explanatory power to be more fundamental than the desiderata specific to conceptual engineering.

First, let’s look at the function/accident distinction problem. The function/accident distinction is a minimal condition every theory of function has to abide by. The distinction dictates that not all effects of explanandum (a trait, or in our context, a concept) are its function; some effects are side-effects or accidental effects. For instance, the function of the heart is to pump blood, but a heart also emits sounds. An adequate theory of function ought to draw the line between the function of an explanandum and its side effects. In the case of the heart, the theory should not predict that the function of the heart is to produce heart sounds. The goal of a theory of function is to propose a well-drawn function/accident distinction.

How do forward-looking theories fare in regard to this distinction? A simple forward-looking theory like Canfield’s postulates that a function of a trait t in system s is to do f just in the case that t does f and that f is done is useful to s (1963, p. 290). However, it has been criticized as not being able to draw a sharp function/accident distinction (for example, Frankfurt and Poole (1966)). The problem is that side effects sometimes have beneficial consequences.^23^ For instance, heart sounds may lead to a higher survival likelihood for a person since the invention of stethoscopes. Alternatively, the fact that noses can support glasses may also help humans survive better, for example, because they can successfully detect deadly mold on their food with their help. Therefore, a simple good-contribution theory will not do.^24^

Is this also a problem for concept function accounts that build on the Usefulness Thesis, that is accounts that posit that a constitutive feature of a concept’s function is its usefulness? There are good reasons why the answer to this question is yes: Even with the proviso that we do not focus on individual uses of a concept but its possible uses across different types of situations that provoke our concern, the problem of overreach rears its head. Consider, an abstract concept, such as TRUTH. It can be used to satisfy the concerns of scientists to track natural truths, it can be used to discredit your opponents by accusing them of misrepresenting the truth, it can be used to build formal logical systems, it can be used to predict the mental states of others by tracking what they hold true, a use can satisfy the concern to build philosophical theories (e.g. build a JTB analysis of KNOWLEDGE), it can satisfy the concern to validate someone’s experience, it can be an object of aesthetic beauty in poetic novels, it can also be used to justify oppressive structures, etc. The problem is that some of these effects are more and less plausible candidates for the function of a concept but forward-looking theories are unable to adjudicate between these ascriptions. They all turn out to be the function of a concept. This fact becomes even odder when we consider that they also allow for contradictory ascriptions. A concept C may be used to satisfy N by person A, but at the same time that C is used to satisfy not-N for person B. For example, race concepts will probably produce this effect. A may use them to satisfy their white supremacist concerns, while person B might use them to advocate against white supremacy. The mere mapping of concept use and the satisfaction of some concern does not explain for instance how some of these contributions are derivative of more fundamental ones. The mapping does provide interesting insights into an aspect of the concept, but what exactly about this explanation will help conceptual engineering projects? Forward-looking theories include too much in their explanations, so they end up too broad to be useful.

The same problem arises for Thomasson’s Absence-Considerations Account. If some side effects of a concept are useful and we explore what useful features would be lacking without said concept, then our analysis would not be able to draw a line between beneficial side effects and function. It is not solely the function that is useful to concept users. The insight from the biological function literature that the entirety of useful effects of an explanandum does not constitute the function still applies to both accounts.

What about the other adequacy condition, the normativity of function? Here, too, forward-looking theories arguably fail. The normativity of function condition requires that function theories explain how an explanandum can have a function that it is unable to fulfill-what it should do in what circumstance. Critics complain that since they define the function of an explanandum as the effects that are relevant for its future, these theories muddle the distinctions between duly fulfilling its function, malfunctioning versus being disadvantaged, and non-functioning traits (Garson, 2007, 2016).^25^

Can the Concern-Satisfaction account explain how a concept could have a function that it is not able to fulfill? Prima facie, the answer is yes. Again, the theory has a ‘propitious-circumstances’condition built in. The account does not promise that the use of a concept should always satisfy our concerns. Concepts would turn out to be magical tools otherwise. Only “when circumstances concur” can we hope that concepts will be able to fulfill their function, that is “to satisfy a concern of type C by producing E” (Queloz, 2022, p. 19).

However, how should we understand‘propitious circumstances’, and does it help to explain when a concept malfunctions? All function theories are conditional on a‘normalcy condition’ (Garson, 2016, p. 71). The functions of biological traits are only said to have a function in their normal or natural environment. For example, the beaver’s urge to stop running water and to build dams is advantageous in large natural habitats because it provides them with a home and reshapes the environment to facilitate this. However, when beavers settle in farmlands or even cities, this trait does not produce the same effects as in the environment they evolved in. The trait is non-functional or even disadvantageous. Here, the idea of a natural environment can be specified as the particular evolution environment, so the normalcy condition does a lot of explanatory work.

But what does it mean to say that some concept application is propitious over and above saying that one was able to apply a concept successfully? Take again Queloz’s example of the concept of FIRE. The narrative goes as follows: Humans have a certain concern, namely to recognize fire in order not to burn themselves. This gives them reasons for using a concept, that is to use the concept FIRE when there is fire. By using FIRE in the presence of fire, they are better able to escape dangerous situations involving it, so they change their behavior and their beliefs. This story seems to imply that under propitious circumstances, the effect of the concept application of FIRE is to avoid fire. If a person is interested in fire and does not avoid it, does it follow that the concept has *not been used under propitious circumstances*? Or does it have two functions: to avoid and to advertise fire? Or is one of them the right description and the other false? It is left unclear how to deal with this analysis impasse by only considering concern and need satisfaction. The more important question is: what does the propitious circumstance proviso contribute to the function ascription? It does not seem to influence the function/accident distinction, unlike in the case of biological traits because a normalcy condition is not the same as a propitious condition. The former can be reduced to a specific environment and history, while the latter is not specified according to something else. The propitious circumstances condition merely seems to dodge the demand that it must satisfy the concern in any given situation. This is essentially a circularity problem: Effect E is the function of X iff X produces E under propitious circumstances (plus other conditions), if X does not produce E (plus other conditions), then the circumstances are not propitious.^26^ This explanation of function is hopelessly unhelpful. The propitious circumstance condition turns out to be vacuous as it stands. The idea of ‘propitious’ smuggles in the idea of optimal functioning that is not equivalent to the‘natural environment’that is usually invoked in functional explanation. However, the idea of optimal functioning and when something in fact works is left open. To be clear, I am not arguing that this is a matter of intuition: the argument is not that according to some intuition about concept functions, one ascription is the function while another is intuitively implausible and the account does not explain this intuition. My argument is about explanatory power: the Concern-Satisfaction Theory is unable to give reasonably determinate function ascriptions. The propitious circumstance-condition is vacuous and its role is precisely to draw on our intuitions about what we want the function of a concept to be. Forward-looking theories of function are explanatorily unstable.

## Foward-Looking Functions, Usefulness and Conceptual Critique

One could argue that this is not a problem for accounts that build on (UT) because my ‘instability’ is just ‘context-sensitivity’ and that is a perk and not a disadvantage. This is the route that especially Riggs (2021) and Jorem (2022) take. Riggs distinguishes between substantive and deflated theories. Substantive theories are ones that are “not highly context-dependent or interest-relative, so the function of a concept doesn’t change across contexts just because the people in that context are using it differently, or because the people evaluating that concept have different interests” and deflated are the opposite, meaning highly context-dependent and interest-relative (Riggs, 2021, p. 11576). He argues that substantive theories cannot explain the desiderata that are posed on account of concept functions. I will not rehearse those here; it is only necessary to note that Riggs thinks that we should opt for these highly context-dependent functions that do not give us invariable concept function descriptions because it is more important to remain neutral to what will end up important for a conceptual engineering project. The idea is essentially that we should not rule out a set of capacities and effects of a concept from the get-go because they could be important for the project in question. Perhaps context-sensitivity is not just not an issue, but a virtue for conceptual engineering. I will come back to this argument further below.

Even though I do think that some stability is necessary for concept functions to be helpful in conceptual engineering projects based on my explanatory power argument above, I also believe that accounts that build on (UT) fall prey to another major problem, namely that these accounts are not sufficiently suited to conceptual critique (see also (von Samson, 2024)). Given that conceptual engineering is in the business of criticizing concepts and that concept functions are supposedly the most promising tool to do so, whether or not they lend themselves to criticizing particular concepts should be a more important desideratum than whether they, for instance, explain topic continuity. The very promise of identifying a function of something is knowing what it is ‘supposed to do’, thus providing the conceptual engineer with standards of whether it is satisfying that purpose (most efficiently). This is the very first desideratum of ‘Assessment of concept efficiency’. Since this desideratum is, so to speak, built into the idea of a function, it is the most fundamental one of the three desiderata identified in the conceptual engineering literature. In this section, I want to expand on this desideratum. I will distinguish between two kinds of possible criticism, revision and replacement critiques, and show how forward-looking theories built on the Usefulness Thesis can only capture one.

What do I mean when we say that we should be able to criticize concepts? It is fairly accepted that concepts enable us to understand the world by structuring it by means of application principles that systematically include certain things into its extension and exclude other things. In other words, concepts are general, concepts do this by some sort of application conditions, something, such as truth conditions, rules for application, necessary and sufficient conditions, or psychological entities like exemplars, prototypes, or theories, enable us to specify when a concept applies and when it does not Machery (2009); Isaac (2023). If we think of our conceptual practices, i.e. distinguishing between Cs and not-Cs, as a sort of structuring that buttresses our social practices and institutions, we should pay a lot of attention to our concepts and the distinctions that come with them and whether they are fitting or appropriate. Some distinctions are very helpful, for example, distinguishing between DEATH and not-DEATH, while others are problematic, such as distinguishing between NORMAL and not-NORMAL (in the normative sense), and building our conceptual practices around them. In the former case, the concept of DEATH allows us to regulate when we should stop medical treatment, when to inform the family, when to initiate the grieving process, etc. In the latter case, the social practices built on those concepts might bar a ‘non-normal’ person from invitations to participate in social events, from having friends or receiving job offers, etc. As common as this practice seems, this distinction glosses over what kind of people get labeled ‘not-normal’, often it is the people who are mentally ill, immigrants, etc. Basing a social practice, for example, who gets the job and who does not, on this distinction has serious moral shortcomings. Criticizing a concept is closely related to these types of criticism. With these concepts, I take it that it is uncontroversial that an account of concept functions should reveal them as ideologically flawed. Conceptual functions are supposedly the best candidates to explain when a concept should be criticized because they ought to make apparent what a concept’s purpose is. To be useful for conceptual engineering, they should reveal something about the concept that is non-trivial, otherwise, they are no help to conceptual engineers because they would have no guiding power.

How do accounts based on (UT) fare with concept criticism? I think that they are unsuitable for concept criticisms exactly because they rely on the Usefulness Thesis. To see this, we need to consider some basic features of critique. I will use ‘critique’ in a neutral sense, meaning that a critique can be positive or negative.^27^ Critiquing a phenomenon P is assessing it and possibly coming to a positive or a negative judgment about it. Criticism is a negative judgment of P. Furthermore, critiquing a phenomenon P has normative standards, so there is such a thing as good and bad critique. For example, a few general standards for good critique are: you are being fair about P, i.e. not critiquing a straw man version of P; the standards you are applying to P are apt, i.e. you are not judging a children’s book with the same standards as the great American novel; the critique is balanced, i.e. not overly focused on some parts while ignoring others; the critique is reason-giving, i.e. the critique relayed in such a way that makes it comprehensible to critique-recipients; etc. These are just some of the standards that distinguish a good critique from a bad one and even though they are not exhaustive, they should help make clear when criticism is made difficult. Why is this important? Concept functions have been introduced in the debate to enable us to critique our concepts, so getting clear on what we can expect from concept functions is necessary to assess whether an account of concept functions meets these expectations.

Now that we have a general understanding of critique, let’s return to the accounts of concept functions that build on (UT) and why they are not helpful with concept criticism. A concept critique takes as its basis some sort of understanding of the concept in question. The proposal at hand is that (part of) the basis for conceptual critique is the concept’s function. In other words, the concept’s function is the groundwork on which we base our critique. What sort of understanding of a concept do accounts that are based on (UT) offer? They pick out the capacities and effects of a concept that are useful, that is, they are models that abstract from infelicitous effects and highlight the contribution of a concept that helps us to do things effectively and efficiently or to satisfy our concerns and needs. Thus described, it should be easy now to see how these accounts distort a concept’s function.

Abstracting from all the effects that are either infelicitous, that do not let us do things effectively and efficiently, or that do not satisfy our concerns and needs, are *the effects that usually critique aims to take into account*. If wearing a type of shoe consistently leads to misalignment of the foot and thus to foot deformities, then explaining why we go in for a shoe such as this, for example, to keep our feet warm and dry, and potentially because these kinds of shoes are stylish, seems to be missing the point. This is exactly not the kind of account we need for conceptual engineering purposes if we are interested in concept critique. It abstracts from all the features, contributions, capacities, and effects that would help us to assess when a revision was an amelioration or not. It gives us only reasons *to* engage in a practice and does not give us any reasons *not to* engage in it by itself.

Let’s not be hasty—most forward-looking theorists do not deny the importance of concept critique and have a story to tell about it. How does Queloz suggest that concept critique works?^28^ He proposes a method that should help us to get at a concept’s function, namely a so-called ‘pragmatic genealogy’ (Queloz, 2021). Very roughly, a pragmatic genealogy is a ‘dynamic model’ that tells a historic-fictional narrative of how and why a conceptual practice might have come about. We start out from a very abstract proto-practice that only draws on basic, structural human needs and add socio-local needs to the model as we develop it to come closer to our current practice (Queloz, 2021, p. 51). The idea is something like this: we ought to know the normative standard for a conceptual practice, i.e. what it means when all goes right, then we can measure it against its factual state; if the actual state does not concord with the ideal standard or when we, upon reflection, decide that we no longer identify with the concern or need, then we have reason to criticize or ameliorate said practice. An ideal model of the practice is then helpful for conceptual critique.

Modeling practices have a long-standing tradition in philosophy, but there have been compelling criticisms made of this method advanced by Mills (2005).^29^ He distinguishes between two senses of an ideal model (based on Rawls (1971)) (Mills, 2005, p. 166):

*(1) ideal-as-descriptive:* Imagine some phenomenon of the natural or social world, P. Then an ideal in this sense is a representation of P. One kind of representation purports to be descriptive of P’s crucial aspects (its essential nature) and how it actually works (its basic dynamic).

*(2) ideal-as-idealized:* Since a model is not coincident with what it is modeling, of course, an ideal-as-descriptive model necessarily has to abstract away from certain features of P. So one will make simplifying assumptions, based on what one takes the most important features of P to be, and include certain features while omitting others: this will produce a schematized picture of the actual workings and actual nature of P. But for certain P (not all), it will also be possible to produce an idealized model, an exemplar, of what an ideal P should be like.

To illustrate, consider an ideal-as-descriptive model in sociology. This kind of model abstracts certain ideal features to explain complex phenomena, such as the flow of money during major events like the Olympics and its impact on the local economy. The model’s purpose is descriptive; it aims to represent the workings of the system rather than assess whether it functions ‘properly’ or ‘optimally’. In contrast, an ideal-as-idealized model is a *normative model* of x, i.e. how x should be. Take as an example the blueprint of a specific bridge. Even if the bridge has collapsed, the model gives us an abstraction not of how the bridge has in reality behaved, but of how it functions optimally. Of course, both of these methods have their explanatory place, but the question is: which one is the appropriate one for the purposes at hand? It seems obvious that for conceptual engineering, forward-looking theorists opt for ideal-as-idealized models.

There is a significant problem with pragmatic genealogies as ideal-as-idealized models. They tend to prioritize reasons *for* a practice while relegating reasons *against* it to a secondary status. By modeling a practice as working ideally, these genealogies operate under the assumption that it is possible to abstract away from negative aspects and focus solely on its positive features. Furthermore, pragmatic genealogies often rely on the concept of supposedly basic structural needs shared by all humans, framing the discussion in a way that connects our concepts to perceived universal needs. This framing can inadvertently sway justifications in favor of specific conceptual practices from the outset. When there is a need to de-idealize a practice, critiques are then limited to local, historically contingent reasons. In contrast, justifications draw on alleged aspects of human nature. The result of assessing a concept via pragmatic genealogy is an imbalanced critique (von Samson, 2024).

A related issue is that if we consider ideological concepts like NORMAL, PRIMITIVE, or CIVILIZED, a pragmatic genealogy would likely depict them as non-ideological. Instead, it would recount a (hi)story about how humans have specific concerns and needs that lead us to categorize others based on certain conditions necessary to fulfill basic needs. This approach militates against a balanced critique, as it presumes what counts as the optimal conceptual practice. The question of “optimal according to whom?” remains open, as previously discussed. In this case, it is clear that those who introduce these concepts-such as colonizers for PRIMITIVE and CIVILIZED-are the ones who benefit from such narratives. Furthermore, since pragmatic genealogies assess concepts based on their ability to meet basic human needs, they rely on assumptions about these needs, like the belief that individuals naturally favor their family or in-group. These assumptions are not beyond scrutiny and can perpetuate implicit biases about what is essential for humanity, thus they make it harder to regonize, even upon reflection, when a concern or need is no longer ours. A radically critical conceptual engineer might challenge not only the genealogical framework but also the very concepts used to define these needs, such as FAMILY or COMMUNITY. Consequently, pragmatic genealogies risk promoting a distorted understanding of what constitutes a ‘civilized’ society. Ultimately, pragmatic genealogies, qua ideal-as-idealized model, incorporate specific values that dictate when a conceptual practice is deemed to function ideally, limiting the potential for fair critique. While pragmatic genealogy can serve as a useful justificatory method-similar to Williams (2004)’s efforts to counter skepticism, relativism, and postmodernism-it falls short as a tool for critical analysis when the aim is to explore a concept’s function in a balanced manner.

A plausible response could be that this still does not show that we should not start our conceptual engineering project with forward-looking functions. There is nothing wrong with considering the optimal functioning of a practice *first* if we are still able to balance positive and negative considerations before we engineer. Forward-looking function theorists mention this strategy because of course sometimes a conceptual practice has many negative effects that outweigh the positive ones (Queloz, 2021; Köhler & Veluwenkamp, 2024). Forward-looking theories try to first isolate the forward-looking function, which will help us to determine standards for when a concept goes right, and then *secondly* take into account other effects, including those that speak against said practice. We can then balance the reasons for and the reasons against it. It is not necessary to stick with just positive effects. However, again in pragmatic genealogies, there is a structural and systematic bias toward justifying and against criticizing the conceptual practice since all negative considerations are *per definitionem* not part of the function of a concept according to forward-looking theories. This is a dubious claim when we consider concepts like CIVILIZED RACES. This is however not all.

In this context, we should distinguish between two types of critiques that conceptual engineers are interested in, namely (1) revision critiques and (2) replacement critiques.^30^ The former critiques a concept for not being efficient enough, i.e. if the concept RAPE was supposed to refer to all morally objectionable acts of sexual violence, then it didn’t do so when it excluded marital rape. This is connected to the first desideratum for accounts of concept functions ‘The assessment of concept efficiency’. There is however a second kind that frequently appears in the conceptual engineering literature, namely that sometimes we want to *replace or abandon* a concept (not just revise it) (Nado, 2019). For instance, the concept RACE is regularly argued to be too flawed to allow of amelioration. The desideratum that concept functions should help us assess concept efficiency is too narrow for cases such as these. For pragmatic genealogies only help us to assess, given the best possible version of the practice, whether that practice is done most efficiently. Thus, this impedes more fundamental critiques that seek to replace certain conceptual practices. Including the option of replacing certain conceptual practices is important because our concepts can get out of our control: we can design a concept with a specific design function Simion and Kelp (2019), and it may develop ‘a life of its own’. For instance, Haslanger’s redefinition of ‘woman’ was intended to support feminist theorizing. But it was noted that this redefinition could inadvertently exclude trans women-an unintended but significant consequence of adopting that concept (Jenkins, 2016). This demonstrates that it is insufficient to merely identify the need or concern a concept is intended to address; we must also remain attentive to unforeseen outcomes in its practical application. Mapping only how a concept satisfies one of our concerns and needs cannot capture the broader functioning of a concept and specifically what kind of effects it has that might outweigh the ‘optimal functioning’. The hope of finding the most useful account of concept functions is that revealing the function of a concept will help us decide whether we want to retain or abandon this conceptual practice, depending on a neutral assessment. However, concept function theories that are built on forward-looking considerations like the Usefulness Thesis only cater to revision critiques, because they already assume that practice is positive and connected to our needs and concerns. The conceptual engineers that wish to criticize a practice fight an *uphill battle* against the framing of a conceptual practice as being directly connected to our basic human needs. If the concept PRIMITIVE for example satisfies the need to create a strong economy and ‘to help’ ‘underdeveloped’ cultures, then arguing against such ‘noble’ reasons is difficult, to say the least. And these were among the reasons that were given when these social practices emerged.^31^ Of course, it should be conceded that pragmatic genealogies *do not* make critique entirely impossible. In the PRIMITIVE case, if we only question certain assumptions made by the story, we can safely arrive at a helpful criticism. In hindsight, the narrative cannot fool us anymore because a lot of work has been done to disentangle the practice of distinguishing between primitive and not-primitive cultures, but that was only possible *despite* the justification of it not *because* of it. The argument from distortion still stands, and the question is why should we start out from the justification, i.e. the ideal-as-idealized model, and not from a historical-local description, ideal-as-descriptive model, of the practice? The correct stance is: we should not.

What does this mean for pragmatic genealogies? That they rule out criticism altogether? That would go too far; pragmatic genealogies can identify important needs and concerns that are satisfied by the concept and help us assess how well those are served (revision criticism). The place where pragmatic genealogies fail us is at the main desideratum ‘assessment of concept efficiency’ if by that we ought to mean ‘comprehensive assessment’, i.e. assessing whether we should revise and whether we should replace or abandon. This means they fail us when we want to know whether the conceptual practice is a good one at all (replacement criticism), that is, whether we want to/should continue the practice. Because pragmatic genealogies are built to pick out positive contributions of our concepts in a practice, they will always latently pull us in the direction to ‘just make the practice more efficient’ and not question the practice itself. In other words, pragmatic genealogies can only help us to answer whether we want to revise a concept, but not with fundamental critiques and the question of whether we should replace or abandon a concept. Even more importantly, the argument is not that we never want or need to know the forward-looking function (concern-satifaction, normative function or its absence-consideration), but that it does not play the desired theoretical and practical role for conceptual engineering projects. The main desideratum, remember, was that it allows us to assess a concept and concept functions were promised to be the ideal theoretical notion for this purpose. However, the problem is that forward-looking theories identify some relevant effects of a concept and, at the same time, concede that negative considerations are relevant for assessment as well. This means that assessment of a concept is not to be done solely on the basis of concept functions but other conceptual effects (that are systematically excluded to be part of the concept’s function). In other words, forward-looking theories do not fulfill their desired theoretical role, i.e. help conceptual engineers with concept assessment. Their role is, qua ideal-as-idealized model, justificatory in nature (von Samson, 2024).

The purpose of this section was to challenge forward-looking theories of concept functions *based on the Usefulness Thesis*, though some of the arguments extend to all forward-looking theories as touched upon every so often. What defines a theory as forward-looking is that it treats the *positive* contributions of a concept as primarily determinative of its function. Köhler and Veluwenkamp (2024) presents a forward-looking theory of concept functions that is not based on the Usefulness Thesis. Instead, they argue that a concept’s function is defined by what it enables us to do in *normatively* significant ways where the relevant “considerations [are] that count *in favour* of actions, reactions, or attitudes” (Köhler & Veluwenkamp, 2024, p. 407, emphasis added). This approach offers explanatory stability and avoids the issue of an explanatory circle discussed earlier. However, because it identifies positive effects- normative functions that give reasons for the continued use of a concept-it still implicitly assumes that the conceptual practice is worth preserving. The issue is that when a theory of concept functions focuses solely on the effects that should be preserved, it risks downplaying or ignoring the negative effects of a concept’s use, relegating all of them to the status of mere ‘side-effects.’ This oversight can be problematic for conceptual engineering, as it militates against the possibility of comprehensive critique. There is reason to believe that forward-looking theories, by prioritizing concept revision over replacement or abandonment, might lead to premature revisions. Such hasty revisions, without a solid critical foundation, can be costly at best and harmful at worst. While this is not a definitive argument against the possibility of a forward-looking theory that can avoid this issue, it highlights a fundamental challenge that many forward-looking theories face.

There is yet another possible line of defense for these theories that I want to quickly mention. Riggs (2021) distinguishes between two kinds of theories: substantive and deflated. Substantive theories, according to him, are “not highly context-dependent or interest-relative, so the function of a concept does not change across contexts just because the people in that context are using it differently or because the people evaluating that concept have different interests” (Riggs, 2021, p. 11576). On the other hand, deflated notions are the opposite, meaning ascribing a function to x is context-dependent and interest-relative. The issues facing forward-looking theories of concept functions based on the (UT) only arise if we treat pragmatic genealogies and the Absence-Considerations account as substantive theories of function. Riggs however argues that the Concern-Satisfaction Account falls under the deflated category. This implies that when we ascribe a Concern-Satisfaction function to a concept, we’re only making a judgment relevant to a particular context or theoretical interest. Consequently, there would be no single, idealized model of a concept’s function-what counts as part of a concept’s function may vary across contexts or research interests; the same effect could be part of the function in one model and not part of the function in another. The hope is that this reframing of the theory would also potentially help to dispel worries about the account’s pluralism.

However, this response has two significant flaws. First, in relation to my argument about explanatory power, if there’s no stable ground for an explanation, then there’s no meaningful explanation to offer. While Cappelen’s criterion of invariance may be overly rigid, some level of stability is required for explanations to ensure that we are attributing relevant effects to the explanandum, rather than to circumstantial conditions. Second, deflating the account undermines its usefulness for conceptual engineering. Many concepts are likely to encounter a ‘conflict of needs’ problem. A concept may fulfill a need, N, for one community while simultaneously fulfilling the opposite need, not-N, for another community. For instance, race terms can be used by both racists and civil rights advocates, serving opposite goals. In such cases, the Need-Satisfaction account offers no guidance because both sides are equally satisfying their needs. Together, these problems render the account trivial for conceptual engineering if it is a deflated account of concept functions. Deflating the account doesn’t protect it from overly stringent standards; rather, it abandons any hope of making a meaningful contribution to conceptual engineering. Thus, reframing these theories as deflated rather than substantive does not resolve the issues at hand.

I want to end this discussion with only a brief proposal on how pragmatic genealogies could be developed to deliver better explanations for conceptual engineering.^32^ How can we better balance reasons for and against having a conceptual practice? Queloz suggests, drawing on Moore (2006), that pragmatic genealogies start from a ‘disengaged’ (contrasted with ‘engaged’) use (Queloz, 2022, p. 1248). The former is the stance of an ethnograper of a conceptual practice and the latter is that of the practice participant. Queloz suggests that

We thus have to adopt a stance that lies halfway between full abandonment to and full abandonment of the concept. We use the concept in a disengaged way in order to evaluate whether to use it in an engaged way (Queloz, 2022, p. 1249)

As we have seen, merely adopting the stance of an ethnographer-mapping concern-satisfaction and tracking the reasons for a concept’s use based on this mapping-is insufficient. In Queloz’s work, the relationship between conceptual practices and our needs can lead to both reasons for and against maintaining a practice. However, it is not clear why this would be the case without deliberate engagement in criticism.A critical stance must not only assess how a concept satisfies our concerns but also what it fails to capture, map, or explain. A proper critique should not just track reasons for the use of a concept, but also reasons against it. We may thus distinguish between justificatory and critical pragmatic genealogies. Both are giving a fictional (hi)story of a conceptual practice but the former takes the (implicit) justificatory stance towards the practice while the latter takes an explicitly critical stance towards it. The former is in the business of justifying a conceptual practice by telling a story how a concept developed to help satisfy certain concerns or needs, such as the analyses of Craig and Williams. The latter starts from a critical distance to the practice by providing reasons for and against said practice. One could argue that Nietzsche has given a such critical genealogy. Nietzsche criticized our moral concepts while Craig did not criticize our epistemic concepts, not simply because Nietzsche identified a bad practice and Craig a good one. The crux lies not in the concept’s themselves but in the methodology used. Craig’s analysis was implicitly justificatory, whereas Nietzsche’s approach was explicitly critical.

What does Queloz say about critique? Queloz suggests that critique may be a group effort, as a concept can be ‘authoritative’ for one group but not for another, potentially leading to “respectful disagreement” through need and concern pluralism and mutual understanding (Queloz, 2022, p. 1269). However, there is no reason to outsource critique entirely to other concept users with different concerns and needs. Critical analysis demands more than an ethnographer’s stance; it requires a commitment to balanced, systematic critique. If pragmatic genealogies were to track not only Need and Concern-Satisfaction but also all reasons for and against a conceptual practice, the resulting approach would resemble a system function analysis-one that investigates the “mind-independent causal patterns,” which Queloz seeks to set aside. Yet, if we recognize that sometimes concepts can develop a life of their own, these patterns should re-enter the assessment, as they can significantly influence whether a concept remains viable or not.

To conclude this section, I think there is a role for genealogies in conceptual engineering projects. If we openly acknowledge that pragmatic genealogies (and presumably all forward-looking theories of concept functions) are structurally justificatory, they can be used when we are in the business of defending a concept or when we just want to revise a conceptual practice to make it more efficient. One result we cannot hope for is that pragmatic genealogies or other forward-looking theories currently proposed will help us assess whether we should replace a conceptual practice or other farther-reaching conclusions.

## Conclusion

My main goal for this paper was to expound on a recent trend in the concept functions literature, namely theories that build on a version of the Usefulness Thesis. First, I have attempted to explain this trend by, on the one hand, outlining the problem with another rival account, i.e. etiological theories of concept functions, and, on the other hand, by explaining the theories that build on this tenet. Next, I have situated these sorts of theories into a broader taxonomy of the theories of functions. The theories that build on the (UT) are essentially forward-looking theories of function which means they pick out the effects of an explanandum that will affect its future. I have argued that these theories come with their own set of problems, namely they have trouble meeting two adequacy conditions that ensure the explanatory power of a theory of function, that is the function/accident distinction and the normativity of function. Further, I have shown that even if one believes that robust concept functions are not necessary, accounts that build on (UT) impede important types of conceptual critique. To conclude, I support the functional turn in conceptual engineering. By contrast to Riggs (2021) and Jorem (2022), my argument is not meant to show that the idea of concept functions is inconsistent and should be deflated. It is only meant to show that forward-looking functions based on the Usefulness Thesis are not the best candidates. The scope of this paper does not allow me to argue for this in detail, but the argument given in this paper seems to support the system functions account as the most likely to deliver what conceptual engineers are looking for, since its analysis is not biased in identifying only the positive or negative capacities of a concept in a system. The weightiest reason why system function theories are probably the best candidates is that they offer conceptual engineering comprehensive descriptions of a concept’s functioning and thus the most useful empirical basis for a conceptual engineering project.

## Footnotes

1 In the rest of the paper, I will consider the primary target of conceptual engineering projects to be concepts. One reason is that I believe that what is most fruitful for conceptual engineering are shared representational devices that exclude speakers’ meanings from my frame of interest. Therefore, the term ‘representation devices’ is too broad for my purposes. Next, I want to invoke a certain intuitive understanding of the phenomenon I have in mind. I do not intend to use ‘concepts’ in a theory-laden way, such as specific psychological entities (Machery, 2009) or abstract entities (Frege, 1884, 1918). I understand concepts as (1) what is expressed by words, specifically predicates, (2) as public, and (3) general (see (Glock, 2009).

2 None of these authors subscribes to every desideratum explicitly.

3 I will use capitals to indicate when I am talking about concepts instead of words.

4 This is ambiguous between whether ‘determinative’ is a matter of epistemology or ontology of function. I will assume that we are talking about the ontology of concept functions because we want to identify the contribution of a concept that is its function. However, there is an interesting argument to be made that the usefulness of a concept is *evidence* for a contribution being the concept’s function.

5 I will return to these later and propose two alternative desiderata: the function/accident distinction and the normativity of function, which I find more fundamental. I will explain why a theory of function should prioritize these.

6 Presentist theories receive less attention compared to etiological accounts. It is frequently argued that similar objections, aside from the epistemic access problem, also apply to system function theories. Many authors classify etiological and system function theories together under a broader category, often labeled as either (1) ‘orthodox’ or ‘traditional,’ or (2) ‘substantive’ theories, which are contrasted with ‘forward-looking’ or ‘deflated’ theories (Riggs, 2021; Jorem, 2022; Queloz, 2022) I will, therefore, focus solely on etiological theories as representative of this shift away from traditional theories.

7 This is not to say that there has not been any work done to this end. Work in cultural evolution comes closest to what etiologists of concept functions are looking for (see for example Tamariz and Kirby (2016)).

8 Cappelen’s contrast is different: he contrasts ‘variable’ with ‘contextualist’ concept functions. However, because some functions can at least be theoretically (diachronically) continuous but context-dependent, I think it is better to contrast ‘invariantist’ with ‘variantist’. I thank Hanjo Glock for making this point to me.

9 Belleri calls this the ‘epistemic indeterminacy’ of concept functions ((Belleri, 2021), p. 9). She does not constrain her critique to etiological functions, however, the scope of this paper does not allow me to discuss this in depth for other accounts. For now, this remains a problem for theories of concept functions which will need to be explored in the future.

10 See Dutilh Novaes (2020).

11 This excerpt stems from an earlier, unpublished version of Thomasson (2020) that Cappelen cited in his Cappelen (2018) book.

12 Thomasson (2022) has shifted attention from concepts to vocabulary and words. She has given an impressive analysis of how to functionally analyze certain linguistic items in our language, such as modal vocabulary and nominalizations, etc.

13 Queloz (2021) developed two accounts to analyze concept functions, namely the need-satisfaction and now the concern-satisfaction account. Since his concern-satisfaction account seems to be broader than the need-satisfaction account, possibly even encompassing it, I will focus on the new account.

14 To clarify what Queloz considers an effect of a concept: he denies that concepts themselves have effects, but only our dispositions to use concepts which in turn into a practice of using these concepts ((Queloz, 2022), p. 14). This is somewhat cryptic because dispositions are causal factors and do not in and of themselves have causal effects. I will therefore assume that we should analyze the effects of actual concept applications.

15 Queloz does not specify what he means by human concerns. In his earlier writings, he believes that basic human needs, such as safety, communication, cooperation, etc., are the starting point where to begin analyzing our concepts (Queloz, 2021).

16 A much-discussed example in the conceptual engineering literature is the concept of DEATH. Before the invention of medical ventilators ‘death’ meant something like “loss of the capacity to breathe or cessation of the heart pumping blood” (Sarbey, 2016) After the invention of medical ventilators, cessation of breath was not a good indicator of when to cease medical help for a person. So, given the obvious concern not to stop life-support for people who are still alive, ‘death’ now is defined as ‘no measurable activity in the brain’.

17 One could doubt this taxonomy attribution because Queloz’s preferred method to get at c-functions is by performing pragmatic genealogies and genealogies are by their very nature backward-looking. However, pragmatic genealogies do not primarily draw on actual histories of concept use, they explore *fictional* genealogies in order to identify the effects that either do or would satisfy our concerns. Their method is to deduce reasons for concept use from certain natural and social facts about human beings, not to give a history of the concept. Therefore, it would be wrong to classify c-functions as backward-looking functions.

18 This is of course a matter of controversy, but whether you are a metasemantic internalist or externalist, both sides admit to some sort of conceptual control (see Koch (2021) for externalist control).

19 I thank an anonymous reviewer for encouraging me to clarify this point.

20 Because there is a myriad of different forward-looking theories, I will try to stick to the general definition of forward-looking theories, namely theories that predict the function of an explanandum to be the effects that the trait has on the system’s future.

21 For now, I will bracket Thomasson’s proposal because the Concern-Satisfaction account has been developed in more detail.

22 In the last section, I show that desideratum 1 which should help us assess our concept is in tension with desideratum 3 which should guide conceptual engineering.

23 If these beneficial side-effects are co-opted because of their beneficial contribution, they are called ‘exaptations’ (Gould & Vrba, 1982).

24 For forward-looking theories in the philosophy of biology there are a few strategies available not to succumb to this problem. They can (a) appeal to a statistically significant contribution, (b) non-negligibility condition, etc. (Garson, 2016) However, these strategies are implausible for a theory of concept functions, so I will not go further into them.

25 This ‘normativity of function’ objection differs from the objection given in Köhler and Veluwenkamp (2024). Both objections take issue with Queloz’s pluralism. However, they differ in the answer where pluralism creates problems. Köhler and Veluwenkamp argue roughly that the concern-satisfaction account does not best satisfy the normative notion of authority-why we have reasons to adopt a concept-due to its commitment to pluralism. The normativity desideratum discussed here is the demand that since ‘function’ is conceptually related to functional norms, i.e. what something is ‘supposed to do’, the best account of function should help us understand what it is that the explanandum is supposed to do by distinguishing between when the explanandum is functioning ‘properly’, when it is non-functioning or even malfunctioning. This desideratum is thus relevant to explanatory power since it guides the conditions that ought to pick out what the function of the explanandum is.

26 I thank Hanjo Glock for helping me develop this point.

27 This sense is for example in play in ‘critical thinking’ and ‘literary criticism’. I am going to use the word ‘critiquing’ for the neutral sense and ‘criticizing’ for a negative critique.

28 Here again I will mainly focus on Queloz because he gives a longer argument and exploration of how the identification of concept functions could or should work. But I hold that the same issues apply *mutatis mutandis* to the Absence-Considerations Account as well by virtue of relying on the Usefulness Thesis.

29 This point is also made and elaborated upon by von Samson (2024). Their argument goes more in-depth into how pragmatic genealogies are ideological.

30 I thank an anonymous reviewer for pushing me to clarify this point.

31 See especially “The White Man’s Burden: The United States and the Philippine Islands” by Rudyard Kipling (1899) and “The Races of Man: An Outline of Anthropology and Ethnography” by J.C. Nott and George Gliddon (1857).

32 I want to thank Sally Haslanger for pressing me on this.
